# Remission from Depression among Adults with Arthritis: A 12-Year Followup of a Population-Based Study

**DOI:** 10.1155/2014/828965

**Published:** 2014-01-27

**Authors:** Esme Fuller-Thomson, Marla Battiston, Tahany M. Gadalla, Yael Shaked, Ferrah Raza

**Affiliations:** Factor-Inwentash Faculty of Social Work, University of Toronto, Toronto, ON, Canada M5S 1V4

## Abstract

Individuals with arthritis are vulnerable to depression. In this study, we calculated time to remission from depression in a representative community-based sample of depressed Canadians with arthritis who were followed for 12 years. We conducted secondary analysis of a longitudinal panel study, the National Population Health Survey, which was begun in 1994/95 and has included biennial assessment of depression since that time. Our analysis focused on a total of 216 respondents with arthritis who were depressed at baseline. The mean time to remission from depression was calculated using the Kaplan-Meier procedure and compared across categories of each of the potential predictors. The percentage of those no longer screening positive for depression was calculated at two years after baseline. At two years after baseline, 71% of the sample had achieved remission from depression. Time to remission was significantly longer for those depressed adults who were under the age of 55, those who reported more chronic pain at baseline, those with comorbid migraine, and those who experienced childhood physical abuse or parental addictions. These findings highlight the importance of screening for these factors to improve the targeting of interventions to depressed patients with arthritis.

## 1. Introduction 

Arthritis and associated conditions affect one in five adult Americans and approximately half of older adults [1]. Individuals with arthritis are particularly vulnerable to depression [2]. A study of community-dwelling Canadians showed that those with arthritis had twice the odds of depression in comparison to their peers without arthritis [3]. Depression among individuals living with arthritis may have many negative outcomes including increased functional disability [4], increased absenteeism [5], lower adherence to medical recommendations for treatment [6], and a higher prevalence of suicidal ideation in comparison to those with arthritis who are not depressed [3].

The literature identifies several factors which are associated with depression among those diagnosed with arthritis. Individuals with arthritis who are younger [7], single [7], and female [7, 8] and those who are poorer [7] have an increased prevalence of depression. People with higher levels of physical disability and pain [3, 9, 10] as well as individuals who identify daily stressors and limited social support [9] have higher rates of depression. Individuals with arthritis who also suffer from comorbid chronic health conditions have higher odds of depression than those without comorbid conditions [3].

In order to assist health care professionals in providing treatment for those living with both depression and arthritis, it is important to identify factors that are associated with remission from depression in this population. Improved knowledge of these factors can be used to inform outreach and intervention initiatives for the most vulnerable in this group. Due to the sparse research focused on remission among individuals with arthritis, we must draw upon research identifying factors associated with remission from depression in the general population.

Poorer self-rated health [11–13] and limitations in Activities of Daily Living [14] are all associated with lower rates of remission from depression in the general population. Although we are unaware of a study specifically examining whether arthritis is associated with a longer time to remission from depression, several studies have established that the presence of any chronic physical illness is negatively associated with duration of depression [15, 16]. Demographic characteristics such as marriage, gender, and age have been studied to determine their impact on remission from depression. Marriage has been reported to be a protective factor [17, 18] and gender may not be a significant factor in remission from depression [17]. Younger age has been reported to indicate higher remission rates in some studies [15, 17, 19], but younger age at first onset may also predict a more chronic course of depression [20]. Less is reported in the literature about the effect of adverse childhood experiences (e.g., childhood abuse and parental addictions) on remission from depression. Individuals with these experiences have higher rates of depression in general [21]. Recent evidence suggests those who were maltreated in childhood have worse treatment outcomes for depression as adults in comparison to their nonmaltreated peers [22, 23].

There is a clear gap in the literature regarding information on factors associated with remission for those living with arthritis. The aim of this paper is to address this issue by using data from a regionally representative Canadian sample to obtain an understanding of the factors associated with remission from depression amongst those living with arthritis in the community. Identifying these factors is important to inform interventions for those with depression and arthritis.

## 2. Methods

### 2.1. Data Source

The National Population Health Survey (NPHS) is a nationally representative, longitudinal panel study of the Canadian population that began in 1994 [24]. The first wave of the survey was conducted in 1994/1995 and included 17,276 respondents. Every 2 years subsequently, attempts were made to resurvey these respondents. This study draws on information gathered up to 2008/2009, wave 8. In wave 1, the response rate was 83.6%. Of these respondents, 92.8% responded to the survey again in wave 2. By waves 7 and 8, the response rates were 77.0% and 70.7%, respectively [25].

### 2.2. Sample

The sample was comprised of two groups. A total of 138 respondents with arthritis who were aged 18 and older in wave 1 and who were classified as depressed comprised the first group. In order to increase the power of our analyses, we included a second group of 78 adults with arthritis who were not depressed in wave 1 but who were depressed in wave 2 (1996/97). Of the 216 respondents in the combined sample, 169 were women and 47 were men. In order to determine time to remission, each of the groups was followed for 12 years (from wave 1 to wave 7 for group 1 and from wave 2 to wave 8 for group 2).

### 2.3. Measures

The Composite International Diagnostic Interview-Short Form (CIDI-SF) developed by Kessler et al. was used to assess *depression* biennially [26]. The CIDI has excellent interrater reliability and good test-retest reliability and validity [27]. In comparison to the CIDI, the sensitivity and specificity of the CIDI-SF are 89.6% and 93.9%, respectively [27]. Each respondent was asked if he or she had “any of the following long-term conditions that have been diagnosed by a health professional.” On the list was “*Arthritis* or rheumatism.” Respondents were not asked to identify which type of arthritis they had.

Baseline *demographic* characteristics investigated included sex, age group (<55 years or ≥55 years), marital status (married/common law versus single/divorced/separated), and highest level of education (≤high school or >high school).

*Social support* was measured by the respondent endorsing that they had “someone (they) can confide in, or talk to about (their) private feelings or concerns.”

Two adverse childhood experiences were examined: childhood physical abuse and parental addictions. They were assessed by asking the respondent to refer to events that happened in childhood or adolescence, before they left home. Respondents were asked “Were you ever *physically abused* by someone close to you?” and “Did either of your *parents drink or use drugs* so often that it caused problems for the family?”

*Health factors* assessed for physical comorbidities and the presence of pain. Comorbid chronic conditions were identified by the respondent through a checklist of a number of chronic conditions “that had lasted or are expected to last six months or more” and that had been “diagnosed by a health professional.” We included the assessment for two common chronic conditions: back pain and migraines. General *pain* was measured by the respondent's endorsement of the question “Are you usually free of pain and discomfort?”

*Physical activity level* was assessed as active, moderate, and inactive based on a sum of daily recreational physical activities lasting more than 15 minutes.

### 2.4. Analyses

We investigated remission using survival analysis, based on a biennial measurement of depression over a 12-year observation period. This period started in 1994/1995 and ended in 2006/2007 for the 178 individuals with arthritis who were depressed at wave 1. For the 78 individuals with arthritis who were not depressed at time 1 but were depressed at time 2, the assessment period began in 1996/1997 and ended 2008/2009. Censoring occurred if the respondent died or was lost to followup before remitting or if they were consistently depressed in each wave until the end of the 12-year observation period. Remission was defined at the wave in which the respondent no longer met the criteria for depression. The percentage of those no longer screening positive for depression was calculated at 2 years after baseline. The mean time to remission was generated using the Kaplan-Meier procedure and compared across categories of each of the potential predictors. Analyses were conducted using SPSS Version 18.

## 3. Results

Within two years of baseline, 70.8% of depressed individuals with arthritis in this study had achieved full remission. For men with arthritis, the remission rate was 63.8% and for women it was 72.8% (please see Table 1). The mean time to remission was 3.42 years (95% CI 2.7, 4.2) for males and 3.14 years (95% CI 2.8, 3.5) for females, but this was not significantly different (*P* = 0.42).

Significant differences were found in mean time to remission by age, pain level, migraine, and exposure to childhood physical abuse and parental addictions. Approximately four of five adults with arthritis over age 55 were in remission from depression at two years in comparison to three out of five individuals who were 55 or younger. Approximately, four in five individuals who were usually free from pain at baseline were in remission from depression at two years in comparison to only two-thirds of those who reported at baseline that they usually had pain. Seventy-six percent of those who did not suffer from migraine at baseline were in remission at two years, while only 58.5% of those who suffered from migraines were in remission.

Individuals who reported a history of physical abuse in their childhood had a mean time to remission of almost a year more than those who did not report childhood physical abuse. Approximately three quarters of those who were not physically abused were in remission at two years in comparison to 59% of those who reported childhood physical abuse. Individuals exposed to parental addictions also had a lower prevalence of remission by two years in comparison to those without addicted parents (62.2% versus 72.3%).

## 4. Discussion 

The findings of this study highlight a positive prognosis for this population. Although individuals with arthritis are more vulnerable to depression, 71% of depressed community dwelling individuals with arthritis in this representative study experienced full remission from depressive symptoms within two years. In the general population it has been found that after two years 80-81% of clinical patients are in remission from depression symptoms and that after 10 to 15 years this number rises to 93-94% of patients [28]. This is a hopeful message, especially in light of the serious consequences of depression to both the individual and society.

This study also provides important information about factors that may impact the course of depression for those experiencing both arthritis and depressive symptoms. Not surprising, those who were not usually free of pain at baseline had a longer time to remission than those who were usually free of pain. This is consistent with the literature regarding pain and depression in the general population where it has been reported that the presence of pain can lead to worse depression outcomes [17, 29, 30]. However, pain is a complicated issue in this population, as the severity of pain associated with arthritis may lead to common misconceptions regarding expectations and outcomes. It is possible that some clinicians and the patients themselves may consider that in the context of pain, depressive symptoms are inevitable with the expectation that they must be tolerated. The result of these misconceptions leads to the perception that treating the depression will not be effective, or even appropriate [31]. Despite these beliefs, studies have reported positive outcomes and effective depression treatment strategies for individuals with arthritis [32]. In one study where enhanced depression care management was employed in comparison to patients receiving usual care, a range of positive outcomes was evident after one year including improved arthritis related pain and functional outcomes, fewer depressive symptoms, as well as better general health status, and overall quality of life [33]. Focused pain management strategies may also be important for more effective depression treatment [34]. Although the presence of pain in those with arthritis is common, our findings underline that the patient's experience of the intrusiveness and consistency of that pain is an important factor to monitor and strive to ameliorate.

Comorbid migraines, in this sample of depressed individuals with arthritis, were found to have a strong influence on the course of depression. Migraines are associated with depression in the general population [35]. Both migraine and depression uniquely impact work productivity [36] and lead to increased medical costs [37, 38]. Our finding that comorbid migraines increase the time to remission from depression among those with arthritis speaks to the importance of screening for migraines in this population.

Childhood adversities appear to cast a long shadow on physical and mental health outcomes for those who experience childhood physical abuse and parental addictions [39]. Children of parents with addiction issues were found to have a higher number of other adverse experiences in childhood; subsequent adult depression in this group was found to be largely a result of these adverse childhood experiences [40]. Childhood maltreatment may impact the onset of depression as it has been found to increase the risk for adolescents and young adults in developing depression [41]. A meta-analysis of 16 epidemiological studies and 10 clinical trials on childhood maltreatment and depression [23] found that adverse childhood experiences, such as abuse and family violence, were associated with an increased risk of developing persistent or recurrent depression and worse treatment response [23]. Clearly, individuals who had experienced childhood physical abuse in our sample of depressed individuals with arthritis show a similar pattern of prolonged time to recovery. Our findings that experiences of childhood abuse and having had an addicted parent lead to longer times to remission indicate that adverse childhood experiences are still significant even in the presence of arthritis and comorbid health conditions and remain a developmental risk factor for a more chronic course of depression. Although assessment of early childhood adversities has not traditionally been included in clinical interviews with depressed patients, increased screening for these issues and more intensive treatment of depression in those with these early life experiences may be warranted.

Regarding demographic factors, our findings indicate that older adults remit faster than their younger peers. Although this is in contrast to some of the literature [15, 17, 19], other studies suggest that younger age of initial onset of depression may result in more severe symptoms of depression across the life course and account for longer time to remission [20]. In a study of adults over 55, individuals aged 65 and older were more likely to be in remission than those aged 55–64 [13]. Despite the well-established link between female gender and increased prevalence of depression [42], our findings indicate that once depressed, males and females with arthritis have a similar time to remission. It appears that although gender is protective against initial onset of depression [42] it does not play a significant role in the course of remission in those who have succumbed to depression, findings which replicate several recent studies [17, 37].

Findings should be treated with caution because of several important limitations of this study. The community-based sample for the NPHS does not include anyone institutionalized for depression and those experiencing the most severe levels of depression in the community may be less likely to participate in the survey at baseline. Therefore our findings may exclude those individuals with the most severe cases of depression, which may have biased the mean time to remission downward. Furthermore, the NPHS does not include information on the length of time since diagnosis with arthritis. It is probable that those facing a new arthritis diagnosis may be particularly vulnerable to depression, while they come to terms with the disease and its ramifications on their life. We could also not examine the type of arthritis the respondents had, nor disease severity, because this information was not investigated in the NPHS. These factors probably play a role in depressive outcomes. Due to the sampling strategy of the survey, the respondents should be a representative sample of community dwellers with arthritis, probably predominantly comprised of individuals with osteoarthritis or rheumatoid arthritis. Future research should consider the type, severity, and time since diagnosis of arthritis, and the relationship of these factors to depressive symptomatology.

Despite these limitations, there are several important strengths to this study, including its large representative community-based sample of depressed individuals with arthritis rather than a reliance on clinical samples, its unusually long follow-up period of 12 years of data, with biennial measurements of depression, and its examination of the role of early childhood adversities in remission. Thus, the study's findings provide new, representative information to improve the targeting of interventions for depressed community dwelling adults with arthritis.

## 5. Conclusions

Given the complexities of the comorbid conditions of depression and arthritis, it is a very positive finding that seven out of ten individuals in our representative community based study experienced remission from depression within two years. The findings also highlight the importance of considering the depressed individual's experience of pain, the presence of migraines, and experience of early childhood adversities. Given the higher rates of depression among those with arthritis as compared to the general population [2], this information is needed for professionals looking to track the chronicity of depression and to improve the screening and treatment of their particularly vulnerable patients with arthritis who are suffering from depression.

## Figures and Tables

**Table 1:** Mean survival time and percent in remission at 2 years compared across categories of potential demographic, psychosocial, health, and health-behavioural predictors.

	Unweighted Mean to depression free	Lower CI	Upper CI	*P* value	% in remission at 2 years
*Demographics *					
Gender					
Male	3.4	2.7	4.1	0.42	63.8
Female	3.1	2.8	3.5		72.8
Age					
<55	3.6	3.2	4.1	**<0.001**	62.4
55 or older	2.6	2.5	2.9		82.4
Marital status					
Single/separated/divorced	2.9	2.5	3.3	0.092	75.0
Married/common-law	3.5	3.0	4.1		65.2
Education					
HS grad or less	3.1	2.5	3.7	0.35	78.8
Some postsecondary	3.3	2.9	3.7		64.9
*Psychosocial *					
Social support					
Yes confidant	3.2	2.8	3.6	0.95	72.1
No confidant	3.2	2.6	3.7		65.9
*Adverse childhood experiences *					
Childhood physical abuse					
Yes physical abuse	3.9	3.1	4.6		59.3
No physical abuse	2.9	2.6	3.3	**0.013**	74.3
Parental addictions during respondents' childhood					
Yes parental addictions	3.8	2.9	4.6		62.2
No parental addictions	3.1	2.7	3.4	**0.026**	72.3
Self-esteem					
High	2.9	2.4	3.3		77.0
Low	3.4	2.9	3.8	0.13	66.7
*Health *					
Pain					
Usually free from pain	2.7	2.3	3.1	**0.017**	78.7
Not usually free from pain	3.5	3.0	3.9		66.7
Migraine					
Yes	3.9	3.2	4.6	**0.003**	58.5
No	2.9	2.5	3.2		76.2
Back pain					
Yes	3.4	2.9	3.9	0.17	66.3
No	3.0	2.6	3.4		74.8

CI: confidence interval; Values with *P* < 0.05 are highlighted in bold font.
